# Recurrence rate and predictors in non-ischemic reversible bradyarrhythmias

**DOI:** 10.3389/fcvm.2024.1455018

**Published:** 2024-10-09

**Authors:** Sudhanshu Kumar Dwivedi, Akhil Kumar Sharma, Anant Agrawal, Kapil Doomra, Gaurav Kumar Chaudhary, Sharad Chandra, Monika Bhandari, Pravesh Vishwakarma, Akshyaya Pradhan, Rishi Sethi, Ayush Shukla, Abhishek Singh, Safal Safal

**Affiliations:** Department of Cardiology, King George’s Medical University, Lucknow, India

**Keywords:** atrioventricular node dysfunction, sinus node dysfunction, reversible bradyarrhythmia, permanent pacemaker implantation, non-ischemic bradyarrythmia

## Abstract

**Objective:**

Non-ischemic symptomatic reversible bradyarrhythmia is a poorly characterized yet common clinical challenge. This study aimed to assess the incidence and predictors of recurrence and the need for permanent cardiac pacing in patients with non-ischemic symptomatic reversible bradyarrhythmia.

**Methods:**

This prospective single-center study enrolled 124 consecutive adult patients with non-ischemic symptomatic reversible bradyarrhythmia who were followed up for up to 24 months after conservative management during index hospitalization. The primary endpoint was a recurrence of bradyarrhythmia requiring permanent cardiac pacing. The secondary endpoint was a composite of recurrence requiring permanent pacing, readmission, or death. Univariate and multivariate analyses were conducted to determine the predictors of the endpoints.

**Results:**

Sinus node and atrioventricular node diseases were seen in 66.1% and 33.9% of patients, respectively. The most common causes of bradyarrhythmia were negative chronotropic drugs (58.1%) and hyperkalemia (55.6%). Permanent pacing was required in 21.8% of patients after a recurrence. Advanced atrioventricular block at presentation (HR: 6.10, 95% CI: 2.45–15.20, *p* < 0.001) and bifascicular block at discharge (HR: 3.63, 95% CI: 1.58–8.33, *p* = 0.002) during index hospitalization were significant independent predictors of recurrence requiring cardiac pacing.

**Conclusion:**

Non-ischemic symptomatic reversible bradyarrhythmia is associated with a high risk of recurrence. Permanent cardiac pacing should be considered during index hospitalization in patients with advanced atrioventricular block and/or bifascicular block.

## Key message

What is already known on this subject?

• Non-ischemic reversible bradyarrhythmia is a common clinical condition, and correction of underlying reversible etiology is recommended without permanent pacing.

What might this study add?

• There is limited data on the natural history of non-ischemic symptomatic reversible bradyarrhythmia in patients who are discharged without permanent pacing.
• Our study is the first study that exclusively included patients with non-ischemic symptomatic reversible bradyarrhythmia who were discharged without permanent pacing.
• ECG characteristics, advanced atrioventricular block at presentation, and bifascicular block at discharge were independent predictors for the requirement of permanent pacing during follow-up.
• Demographic, clinical, and reversible etiological factors were not found to be predictors of recurrence in patients with non-ischemic symptomatic reversible bradyarrhythmia.

How might this impact on clinical practice?

• Permanent pacing should be considered in patients with reversible bradyarrhythmia who have advanced AV block at presentation and persistent bifascicular block after reversal of bradyarrhythmia, during index hospitalization.

## Introduction

Non-ischemic symptomatic reversible bradyarrhythmia (NSRB) is a common clinical challenge that can lead to hospitalizations (1). Medications and metabolic abnormalities are a common cause of NSRB (1, 2). Depending on the underlying pathology, bradyarrhythmia may recur after the index event (3, 4). Current clinical practice guidelines, such as those for cardiac pacing by the European Society of Cardiology (ESC) and those for bradycardia by the American College of Cardiology/American Heart Association Task Force on Clinical Practice Guidelines and the Heart Rhythm Society, do not recommend pacing for patients with reversible bradyarrhythmia unless additional conditions warrant permanent pacing (5, 6). The natural history of NSRB in patients who are discharged without permanent pacemaker implantation remains unclear. Furthermore, it is also not precisely known if bradyarrhythmia due to reversible causes is purely a cause–effect phenomenon or an indicator of a more severe and progressive underlying conduction system abnormality that manifests with bradyarrhythmia triggered by precipitating factors.

The present study aimed to assess the natural history of NSRB in patients discharged without permanent pacing and to describe the predictors of bradyarrhythmia recurrence requiring permanent pacing.

## Methods

### Study design

Our study was a single-center prospective study conducted between January 2016 and December 2019 at a tertiary care hospital in North India. Consecutive enrolment was attempted during this time, and patients were followed up to the first recurrence of NSRB or for up to 24 months.

The study was conducted in compliance with the International Ethical Guidelines for Biomedical Research Involving Human Subjects, Good Clinical Practice Guidelines, the Declaration of Helsinki, and local laws. All patients provided written informed consent. The study protocol and all subsequent amendments were approved by the Institutional Ethics Committee (0038/Ethics/R. Cell/16, dated 02/01/2016).

### Study population

**Figure 1:**
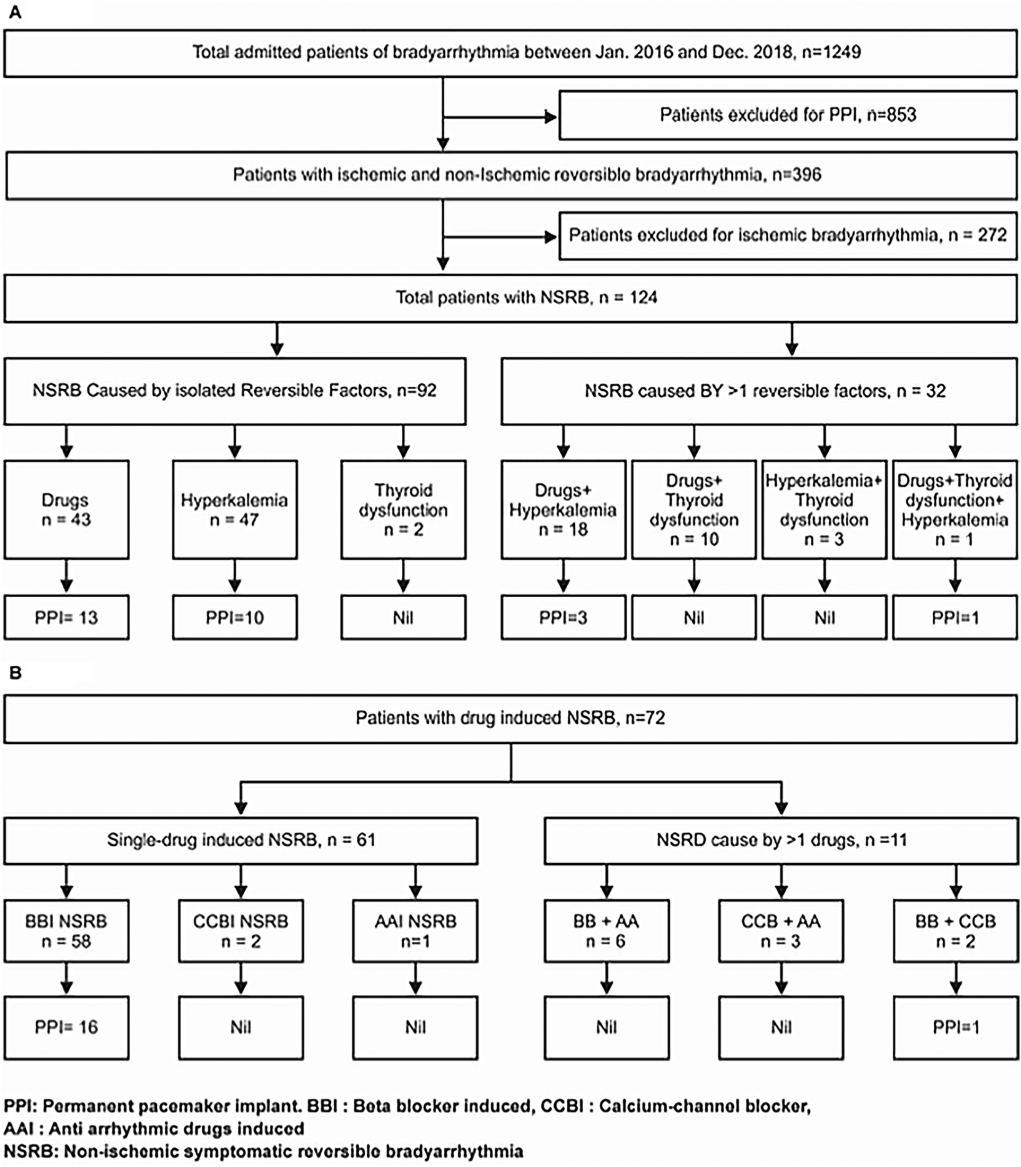
**(A)** Study design and outcomes in patients with patients with non-ischemic symptomatic reversible bradyarrhythmia. **(B)** Outcomes in patients with drug-induced symptomatic reversible bradyarrhythmia.

Adult patients (≥20 years) with a diagnosis of NSRB, who were hospitalized and who reverted to sinus rhythm during hospital stay, were included in the study. Patients were eligible if bradyarrhythmia could be attributed to a prespecified reversible factor such as electrolyte abnormalities, drug-induced, and thyroid dysfunction and if they were discharged without permanent pacing (Figures 1A,B). Potentially reversible etiology was confirmed when the bradyarrhythmia reverted to sinus rhythm following correction of hyperkalemia and thyroid dysfunction or within five half-lives of cessation of culprit drug intake. Patients with ischemic bradyarrhythmia, suspected infective pathology, left ventricular ejection fraction (LVEF) **≤**35%, prior history of bradyarrhythmia, permanent pacing during index hospitalization, and compelling indications of causative drugs and those in whom reversible factors could not be corrected during hospitalization were excluded. In addition, patients with a history of ST segment and non-ST segment elevation myocardial infarction, percutaneous revascularization, coronary artery bypass grafting within 3 months, and ≥20% rise or fall in subsequent values of high-sensitivity troponin T were considered to have ischemic bradyarrhythmia and were excluded.

### Data collection

At admission, a detailed medical history was obtained, and clinical evaluation was performed. Simultaneously, previous hospital records of clinical notes were reviewed to ascertain the secondary causes for NSRB including drugs, electrolyte disturbances, and thyroid disorders and to possibly rule out reflex bradyarrhythmia. Demographic and clinical parameters including age, sex, risk factors (hypertension, diabetes, and renal failure), history of any drug intake at least 1 week prior and continued until 24 h before presentation, and symptoms related to bradyarrhythmia were documented. Laboratory investigations including routine hematology, biochemistry, electrolytes and hormonal assays, and a 12-lead electrocardiogram (ECG) and detailed 2D echocardiography were done at admission. Based on ECG at presentation, patients were divided into two groups: sinus node disease (SND) and atrioventricular node disease (AVND). SND was defined as a sinus rate of <50 bpm and/or sinus pause >3 s, sinus node arrest, or sinoatrial block. AVND was defined as a *P*-wave rate of >50 bpm with second or third-degree block, and advanced atrioventricular (AV) block included patients with 2:1 AV block or higher-degree block (6). Hyperkalemia and renal dysfunction were diagnosed when serum potassium and serum creatinine were ≥5.5 mEq/L and ≥1.5 mg/dl, respectively. Hypothyroidism was diagnosed when the thyroid-stimulating hormone (TSH) level was ≥5 mIU/L, and hyperthyroidism was diagnosed when the TSH level was **≤**1 mIU/L, when patients had a prior diagnosed thyroid dysfunction, or when they were treated for the same. Abnormal ECHO was defined as LVEF >35% and **≤**50%. A final ECG was recorded before hospital discharge. Patients were followed for any symptoms related to bradyarrhythmia, readmission, or death through telephonic interviews every month and during hospital visits every 6 months. A detailed clinical and biochemical evaluation including a 12-lead ECG was performed at every hospital visit or during repeat admission.

### Study endpoints

The primary endpoint was a recurrence of NSRB requiring permanent pacing over a follow-up of 24 months. The secondary endpoint was a composite of recurrence of NSRB requiring permanent pacing, readmission, or resulting in death over a follow-up of 24 months. The clinician's decision for permanent pacing was in accordance with the ESC/European Heart Rhythm Association (EHRA) 2013 cardiac pacing guidelines (5).

### Statistical analysis

The categorical variables were expressed as percentages, and the continuous variables were expressed as mean ± standard deviations (SD). A comparative analysis of SND vs. AVND groups and patients with or without primary or secondary endpoints was performed for demographic features, clinical factors, etiological factors, and ECG characteristics. The categorical variables were compared using the chi-square test or Fisher’s exact test, as applicable. The continuous variables were compared using the Student’s *t*-test or Mann–Whitney *U*-test. Univariate and multivariate Cox regression analyses were performed. The cumulative event-free rates at 24 months were estimated using the Kaplan–Meier method with a log-rank test. A statistical analysis was performed using Statistical Package for Social Science, version 24.0 (SPSS, Inc., Chicago, IL, USA). All *p*-values <0.05 were considered statistically significant.

### Patient and public involvement

All consecutive patients with symptomatic non-ischemic bradyarrhythmia due to reversible etiological factors, who had visited the emergency department, were prospectively enrolled. Eligible patients were asked to participate in the study after the reversal of bradyarrhythmia, and they were planned to be discharged without permanent pacing. All patients gave written informed consent. The public was not involved in the study design, conduct, reporting, or dissemination plans of the research.

## Results

### Demographic and baseline characteristics

A total of 1,249 patients with bradyarrhythmia were admitted during the study period, and 124 consecutive patients with NSRB were included in the analysis (Figure 1A). Of these, 82 (66.1%) patients had SND, and 42 (33.9%) had AVND. The mean age was 64.4 ± 11.6 years (range, 35–89 years), and there were 74 (59.7%) males. The most common presentation of patients with NSRB was syncope (83.1%). Table 1 shows the baseline demographic, biochemical, clinical, and etiological characteristics of all patients. The Echocardiographic details of this study is mentioned in Supplementary Table S1. Renal dysfunction and requirement of temporary pacemaker implantation (TPI) were more common in the AVND group (Table 1). On the other hand, thyroid dysfunction and the presence of combined reversible factors (drugs, hyperkalemia, thyroid dysfunction) were more frequent in the SND group. Although the ventricular rate was comparable in the two groups, features suggestive of underlying infranodal conduction block (QRS ≥120 ms), bundle branch block (BBB), and bifascicular block were significantly more frequent in the AVND group (Table 1).

**Table 1:** Baseline characteristics of patients with non-ischemic symptomatic reversible bradyarrhythmia who were discharged after conservative management.

Patient characteristics	Total (*n* = 124)	SND (*n* = 82)	AVND (*n* = 42)	*p*-value
Age (years)	64.4 ± 11.6	64.5 ± 12.2	64.3 ± 10.3	0.994
Male	74 (59.7%)	46 (56.1%)	28 (66.7%)	0.256
Female	50 (40.3%)	36 (43.9%)	14 (33.3%)	
Risk factors, *n* (%)				
Hypertension	88 (71.0%)	58 (70.7%)	30 (71.4%)	0.936
Diabetes	46 (37.1%)	29 (35.4%)	17 (40.5%)	0.577
Renal dysfunction	81 (65.3%)	49 (59.8%)	32 (76.2%)	0.026
Symptoms, *n* (%)				
Syncope	103 (83.1%)	66 (80.5%)	37 (88.1%)	0.324
Dyspnea	60 (48.4%)	39 (47.6%)	21 (50.0%)	0.797
Fatigue	3 (2.4%)	2 (2.4%)	1 (2.4%)	1
Reversible etiological factors, *n* (%)				
Rate-limiting drugs				
Beta-blockers	66 (53.2%)	42 (51.2%)	24 (57.1%)	0.532
CCBs	7 (5.6%)	6 (7.3%)	1 (2.4%)	0.421
Antiarrhythmic drugs	10 (8.1%)	9 (11.0%)	1 (2.4%)	0.162
Combination of drugs	11 (8.9%)	9 (11%)	2 (4.8%)	0.330
Hyperkalemia	69 (55.6%)	45 (54.9%)	24 (57.1%)	0.810
Hypothyroidism	16 (12.9%)	15 (18.3%)	1 (2.4%)	0.011
Combined reversible factors​	32 (25.8%)	26 (31.7%)	6 (14.3%)	0.036
ECG characteristics at admission, *n* (%)				
Ventricular rate	34.8 ± 8.4	35.0 ± 9	34.5 ± 7.1	0.767
Advanced AV block	38 (30.6%)	4 (4.9%)	34 (81.0%)	<0.001
ECG characteristics at discharge, *n* (%)				
QRS duration <120 ms	56 (45.2%)	45 (54.9%)	11 (26.2%)	0.002
QRS duration ≥120 ms	68 (54.8%)	37 (45.1%)	31 (73.8%)	
BBB^a^	32 (25.8%)	14 (17.1%)	18 (42.9%)	0.002
Bifascicular block	13 (10.5%)	3 (3.7%)	10 (23.8%)	0.001
Biochemical parameters				
Urea (mg/dl)	73.9 ± 41.2	71.4 ± 44.3	78.9 ± 34.2	0.337
Creatinine (mg/dl)	2.6 ± 1.8	2.4 ± 1.7 8	2.9 ± 1.7	0.123
Potassium (meq/L)	5.8 ± 1.5	5.9 ± 1.6	5.67 ± 1.3	0.369
TPI requirement	76 (61.3%)	44 (53.7%)	32 (76.2%)	0.015
Abnormal echo	16 (12.9%)	9 (11.0%)	7 (16.7%)	0.371

Values are presented as mean ± standard deviation for continuous variables.

AVND, atrioventricular node dysfunction; BBB, bundle branch block; CCB, calcium channel blocker; ECG, electrocardiogram; SND, sinus node dysfunction; TPI, temporary pacemaker implant.

All 16 patients with hypothyroidism were hospitalized (average of 13.2 days) till euthyroidism was achieved with hormonal supplements. None of the patients with isolated hypothyroidism required a permanent pacemaker. One patient with hypothyroidism who required a permanent pacemaker after 67 days of enrolment also had hyperkalemia and a history of negative chronotropic drug intake.

^a^ Bundle branch block persisting after resolution of bradycardia during index hospitalization combination drugs included treatment with both beta-blockers and calcium channel blockers. Combined reversible risk factors included the presence of more than one risk factor including drugs, hyperkalemia, and thyroid dysfunction.

### Reversible causes of NSRB

The major reversible factors identified as the cause of bradyarrhythmia were negative chronotropic drugs (*n* = 72; 58.1%) and hyperkalemia (*n* = 69, 55.6%) (Figure 1A). Among the negative chronotropic drugs, beta-blockers (*n* = 66; 53.2%) (Figure 1B) were most frequently associated with bradyarrhythmia, with atenolol (*n* = 35, 53.0%, with a mean dose of 55 ± 14.6 mg) and metoprolol (*n* = 26, 39.4%, with a mean dose of 57.7 ± 2.0 mg) being the most common. The primary indication for beta-blocker use was hypertension (*n* = 51; 77.2%), followed by atrial fibrillation (*n* = 10; 15.2%), with an unknown indication in a minority of cases (*n* = 5; 7.6%). Among patients presenting with hyperkalemia, 61 out of 69 (88.9%) had renal dysfunction, and 15 (22.1%) were on potential hyperkalemic drugs. At the time of presentation, a combination of more than one reversible factor was noted in 32 (25.8%) patients.

### Primary endpoint

**Figure 2:**
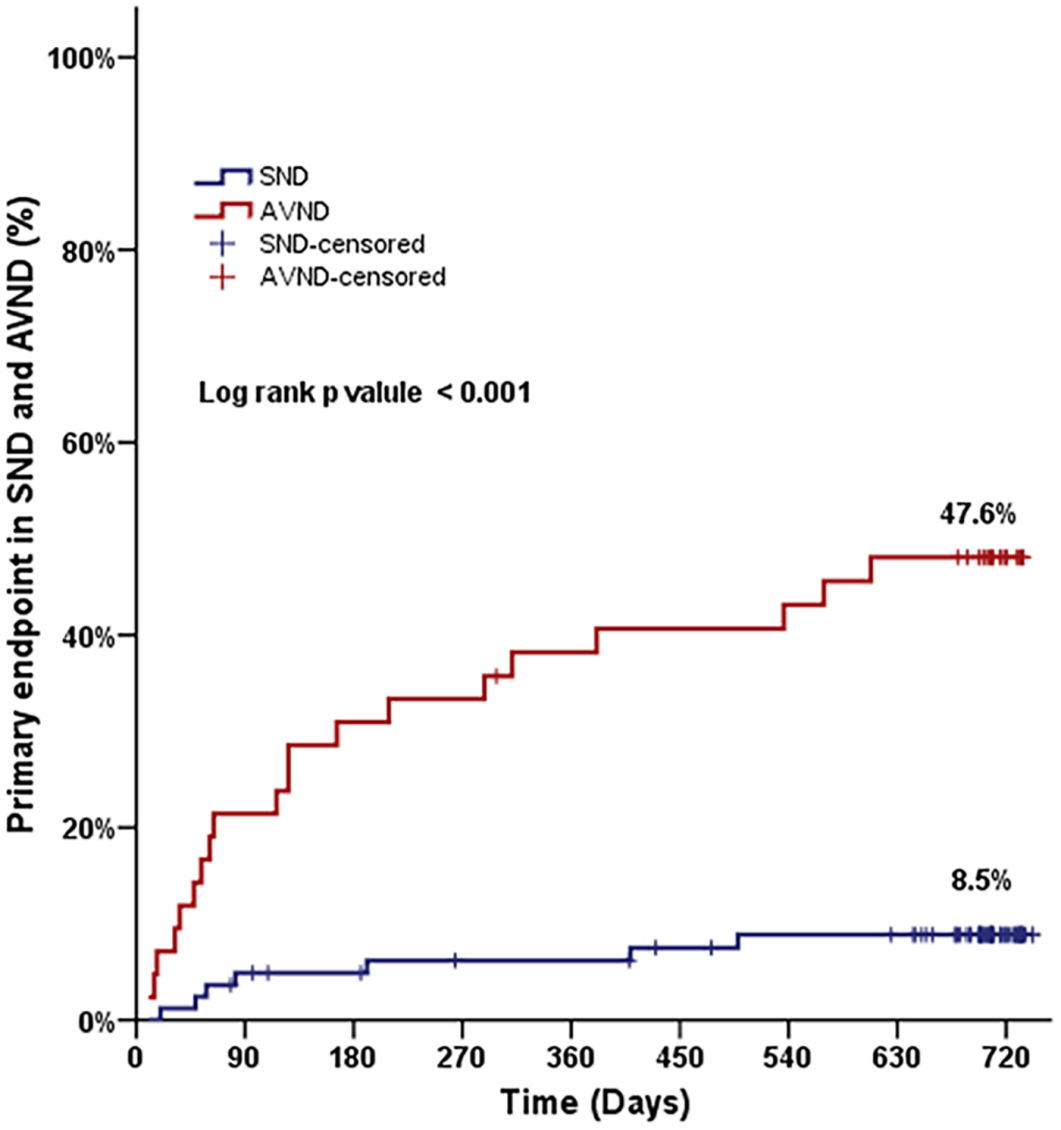
Primary endpoint in patients with sinus node dysfunction and atrioventricular node dysfunction during follow-up.

A total of 27 patients (21.8%) required permanent pacing for recurrence of bradyarrhythmia, over a median follow-up of 700 (range, 10–742) days. None of these patients had an attributable cause for bradycardia at the time of recurrence (Figures 1A,B). Among the patients experiencing recurrence, 18 (14.5%) developed symptomatic advanced AV block. Advanced AV block was detected in seven patients (5.6%) during routine follow-up examinations. Additionally, two patients (1.6%) presented with symptomatic sinus pauses exceeding 3 s in duration. The median time to recurrence requiring permanent pacing was 4 months, and more than half of these patients (17 out of 27; 63.0%) reached the primary endpoint within the first 6 months. When compared to the SND group, more patients in the AVND group (74.1% vs. 25.9%, *p* ≤ 0.001) needed permanent pacing (Figure 2).

Presentation with syncope, a requirement of temporary pacemaker implantation (TPI), and ECG evidence of nodal and infranodal conduction disturbance, such as AVND, QRS duration ≥120 ms, BBB, bifascicular block, and advanced AV block, were significantly more frequent in patients who had a recurrence and required permanent pacing during follow-up, when compared to those who did not have a recurrence (Table 2).

**Table 2:** Key characteristics of patients with non-ischemic symptomatic reversible bradyarrhythmia who attained primary and secondary endpoints.

Patient characteristics	Total (*n* = 124)	Primary endpoint		*p*-value	Secondary endpoint		*p*-value
		Reached (*n* = 27)	Not reached (*n* = 97)		Reached (*n* = 33)	Not reached (*n* = 91)	
Age (years)	64.4 ± 11.6	65.8 ± 9.6	64.0 ± 12.1	0.478	66.2 ± 10.4	63.8 ± 12.0	0.307
Male	74 (59.7%)	18 (66.7%)	56 (57.7%)	0.403	22 (66.7%)	52 (57.1%)	0.339
Female	50 (40.3%)	9 (33.3%)	41 (42.3%)		11 (33.3%)	39 (42.9%)	
Risk factors, *n* (%)							
Hypertension	88 (71.0%)	23 (85.2%)	65 (67.0%)	0.092	25 (75.8%)	63 (69.2%)	0.479
Diabetes	46 (37.1%)	11 (40.7%)	35 (36.1%)	0.658	12 (36.4%)	34 (37.4%)	0.919
Renal dysfunction	81 (65.3%)	16 (59.3%)	65 (67.0%)	0.454	21 (63.6%)	60 (65.9%)	0.854
Symptoms, *n* (%)							
Syncope	103 (83.1%)	26 (96.3%)	77 (79.4%)	0.043	31 (93.9%)	72 (79.1%)	0.052
Dyspnea	60 (48.4%)	9 (33.3%)	51 (52.6%)	0.077	13 (39.4%)	47 (51.6%)	0.228
Fatigue	3 (2.4%)	0 (0.0%)	3 (3.1%)	1	0 (0.0%)	3 (3.3%)	0.564
Reversible etiological factors, *n* (%) Rate-limiting drugs							
Beta-blockers	66 (53.2%)	17 (63.0%)	49 (50.5%)	0.252	19 (57.6%)	47 (51.6%)	0.559
CCBs	7 (5.6%)	1 (3.7%)	6 (6.2%)	1	1 (3.0%)	6 (6.6%)	0.674
Antiarrhythmic drugs	10 (8.1%)	0 (0.0%)	10 (10.3%)	0.116	1 (3.0%)	9 (9.9%)	0.287
Combination of drugs	11 (8.9%)	1 (3.7%)	10 (10.3%)	0.453	1 (3%)	10 (11%)	0.285
Hyperkalemia	69 (55.6%)	14 (51.9%)	55 (56.7%)	0.668	19 (57.6%)	50 (54.9%)	0.840
Hypothyroidism	16 (12.9%)	1 (3.7%)	15 (15.5%)	0.191	2 (6.1%)	14 (15.4%)	0.232
Combined reversible factors	32 (25.8%)	4 (14.8%)	28 (28.9%)	0.213	7 (21.2%)	25 (27.5%)	0.481
ECG characteristics at admission, *n* (%)							
Ventricular rate	34.8 ± 8.4	35.2 ± 8.0	34.7 ± 8.5	0.777	34.8 ± 8. 8	34.8 ± 8.3	0.983
AV node dysfunction	42 (33.9%)	20 (74.1%)	22 (22.7%)	<0.001	20 (60.6%)	22 (24.2%)	<0.001
Sinus node dysfunction	82 (66.1%)	7 (25.9%)	75 (77.3%)		13 (39.4%)	69 (75.8%)	
Advanced AV block	38 (30.6%)	20 (74.1%)	18 (18.6%)	<0.001	20 (60.6%)	18 (19.8%)	<0.001
ECG characteristics at discharge, *n* (%)							
QRS duration < 120 ms	56 (45.2%)	6 (22.2%)	50 (51.5%)	0.007	8 (24.2%)	48 (52.7%)	0.004
QRS duration ≥ 120 ms	68 (54.8%)	21 (77.8%)	47 (48.5%)		25 (75.8%)	43 (47.3%)	
BBB^a^	32 (25.8%)	14 (51.9%)	18 (18.6%)	<0.001	15 (45.5%)	17 (18.7%)	0.003
Bifascicular block	13 (10.5%)	10 (37%)	3 (3.1%)	<0.001	11 (33.3%)	2 (2.2%)	<0.001
Biochemical parameters							
Urea (mg/dl)	73.9 ± 41.2	67.8 ± 28.9	75.6 ± 43.9	0.276	77.5 ± 43.0	72.7 ± 40.7	0.564
Creatinine (mg/dl)	2.6 ± 1.8	2.2 ± 1.1	2.7 ± 1.9	0.221	2.6 ± 1.8	2.6 ± 1.8	0.984
Potassium (meq/L)	5.8 ± 1.5	5.6 ± 1.2	5.9 ± 1.5	0.474	5.8 ± 1.4	5.8 ± 1.5	0.956
TPI requirement	76 (61.3%)	21 (77.8%)	55 (56.7%)	0.047	26 (78.8%)	50 (54.9%)	0.016
Abnormal echo	16 (12.9%)	2 (7.4%)	14 (14.4%)	0.519	4 (12.1%)	12 (13.2%)	1

Values are presented as mean ± standard deviation for continuous variables.

AVND, atrioventricular node dysfunction; BBB, bundle branch block; CCB, calcium channel blocker; ECG, electrocardiogram; SND, sinus node dysfunction; TPI, temporary pacemaker implant.

^a^ Bundle branch block persisting after resolution of bradycardia during index hospitalization.

### Secondary endpoint

Thirty-three (26.6%) patients reached the secondary endpoint [composite of permanent pacing, readmission without permanent pacing, and death in 27 (81.8%), 2 (6.1%), and 4 (12.1%) patients, respectively]. Four patients died during follow-up: one death each due to cerebrovascular accident and renal failure and two (6.05%) deaths due to undetermined causes, potentially associated with cardiac conduction abnormalities. This outcome suggests that more intensive surveillance methods, such as electrophysiological evaluations and continuous ambulatory electrocardiography, could have played a crucial role in preventing these unfortunate events.

ECG characteristics of nodal and infranodal conduction disturbance and requirement of TPI were significantly more frequent in patients who had attained the secondary endpoint when compared to those who did not (Table 2).

### Factors influencing the primary and secondary endpoints

In a univariate analysis, ECG characteristics such as AVND (HR: 6.90, 95% CI: 2.91–16.34, *p* < 0.001) and advanced AV block (HR: 8.42, 95% CI: 3.55–19.97; *p* < 0.001), at presentation, and presence of QRS duration ≥120 ms (HR: 3.34, 95% CI: 1.35–8.27, *p* = 0,009), BBB (HR: 3.52, 95% CI: 1.65–7.50, *p* = 0.001), and bifascicular block (HR: 7.51, 95% CI: 3.41–16.53, *p* < 0.001), at discharge, were significantly associated with NSRB requiring permanent pacing (Table 3). Similar findings were shown for secondary endpoints on univariate analysis (Table 3). Advanced age (≥65 years), male gender, presenting symptoms, and risk factors, such as diabetes, hypertension, or renal dysfunction, were not associated with recurrence. Reversible etiological factors (drugs, hyperkalemia, and thyroid dysfunction), either alone (Table 2) or in combination, were not found to be significantly associated with the recurrence of NSRB (Supplementary Table S2).

**Table 3:** Univariate analysis for association of demographic parameters, ECG characteristics, and reversible etiological factors with endpoints in patients with non-ischemic symptomatic reversible bradyarrhythmia.

Patient characteristics (*n*%)	Primary endpoint					Secondary endpoint		
	Hazard ratio	95% CI		*p*-value	Hazard ratio	95% CI		*p*-value
		Lower	Upper			Lower	Upper	
Age ≥65 years	1.719	0.772	3.828	0.185	1.72	0.834	3.548	0.142
Male gender	1.443	0.648	3.212	0.369	1.452	0.704	2.995	0.313
Hypertension	2.426	0.839	7.017	0.102	1.321	0.596	2.93	0.493
Diabetes	1.126	0.523	2.427	0.762	0.939	0.462	1.909	0.862
Renal dysfunction	0.769	0.353	1.658	0.503	0.929	0.457	1.889	0.839
Syncope	5.906	0.801	43.532	0.081	3.537	0.846	14.784	0.083
Dyspnea	0.499	0.224	1.110	0.088	0.647	0.322	1.3	0.221
Beta-blockers	1.578	0.722	3.447	0.252	1.257	0.63	2.506	0.517
CCBs	0.597	0.081	4.397	0.612	0.481	0.066	3.522	0.471
Antiarrhythmics	0.044	0.000	17.382	0.305	0.341	0.047	2.494	0.289
Hyperkalemia	0.895	0.421	1.906	0.774	1.132	0.567	2.259	0.725
Hypothyroidism	0.246	0.033	1.811	0.168	0.41	0.098	1.713	0.222
AVND (vs. SND)	6.901	2.914	16.342	<0.001	3.753	1.864	7.554	<0.001
Advanced AV block	8.423	3.553	19.972	<0.001	4.598	2.282	9.266	<0.001
QRS duration ≥120 ms	3.335	1.345	8.268	0.009	3.004	1.354	6.664	0.007
BBB^a^	3.523	1.654	7.501	0.001	2.75	1.385	5.46	0.004
Bifascicular block	7.512	3.414	16.531	<0.001	6.506	3.136	13.497	<0.001
Abnormal echo	0.524	0.124	2.213	0.379	0.903	0.317	2.568	0.848
TPI requirement	2.428	0.980	6.019	0.055	2.568	1.122	5.961	0.026

AV, atrioventricular; AVND, atrioventricular node dysfunction; BBB, bundle branch block; CCB, calcium channel blocker; CI, confidence interval; ECG, electrocardiogram; SND, sinus node dysfunction; TPI, temporary pacemaker implant.

^a^ Bundle branch block persisting after resolution of bradycardia during index hospitalization.

A multivariate regression analysis showed that advanced AV block at presentation (HR: 6.10, 95% CI: 2.45–15.20, *p* < 0.001) and bifascicular block at the time of discharge (HR: 3.63, 95% CI: 1.58–8.33, *p* = 0.002) during index hospitalization were the only significant independent predictors of recurrence requiring permanent pacing in patients with NSRB. These parameters were also associated with secondary endpoints (HR: 3.265; 95% CI: 1.534–6.949; *p* = 0.002 and HR: 3.912; 95% CI: 1.785–8.577; *p* = 0.001, respectively). Among patients with advanced AV block at presentation (*n* = 38) and bifascicular block at the time of discharge (*n* = 13), 20 (52.6%) and 10 (76.9%) patients, respectively, were implanted with permanent pacemakers. In a further subanalysis of these two subgroups, patients who received permanent pacing were significantly different from those who did not. The only exception were patients of the bifascicular block subgroup who did not require permanent pacing and had a significantly higher causal reversible etiology in the form of hyperkalemia (Supplementary Table S3).

## Discussion

The present study showed that nearly one-fifth (21.8%) of patients who presented with NSRB and were managed conservatively required permanent pacing over a median follow-up period of 700 days and the majority needed it within the first 6 months of follow-up. Initial presentation with advanced bradyarrhythmia and the presence of bifascicular block at discharge were independent predictors of permanent pacing in multivariate regression analysis. A key strength of our study is that we included only patients who were managed conservatively during index hospitalization and were discharged without permanent pacing. In previous studies, the majority of the patients received permanent pacing during index hospitalization (3, 4). ECG-based stratification of bradyarrhythmia into SND and AVND makes the results of our study relevant even to community cardiology where electrophysiological studies are not routine.

### Incidence of reversible etiological factors

NSRB is caused by several factors including treatment with drugs with negative chronotropic effects, electrolyte abnormalities, thyroid dysfunction, Lyme disease, and rarely hypothermia or inflammatory disorders (7–11). Beta-blockers and CCBs are the most common class of drugs associated with bradycardia (3, 7). In our study, the most common etiologies for NSRB were the use of negative chronotropic drugs (58.1%) followed by hyperkalemia (55.6%). More than one reversible risk factor was seen in about 25% of patients (Table 1). Varying incidence (3%–59%) of hyperkalemia has been reported in patients with reversible bradyarrhythmia (3, 4). In our study, hyperkalemia was seen in 55.6%. This variation can be explained by the variable definition of hyperkalemia and the presence of renal dysfunction in the majority of patients (65.3%) at presentation. Similar to findings in the literature, thyroid dysfunction was seen in 12.9% of patients in our study (8). Unlike Duarte et al. (3), we report a higher rate of more than one etiological factor in patients with bradyarrhythmia (12% vs. 25%). Syncope, a common presentation of non-ischemic bradyarrhythmia, was the most common symptom (>80% of patients; Table 1) in our study (4).

Potentially reversible bradyarrhythmia has a high rate of recurrence. Approximately 25%–56% of patients who experience a resolution of AV block after drug discontinuation are reported to present with a recurrence of bradyarrhythmia (3, 12, 13).

### Incidence of recurrence

Similar to the findings in our study (21.8%), previous studies have reported the need for permanent pacing in 25% of patients who were discharged after conservative management of bradyarrhythmia (3, 12) Farre et al. (4) have reported median time to permanent of 9 months in comparison of 4 months in our study for patients who experience a recurrence bradyarrhythmia. These findings suggest the need for an earlier and timely intervention in such patients.

### Predictors of recurrence

Various ECG characteristics are shown to be predictors for recurrence in bradyarrhythmia. Similar to our study, BBB, AV block, and QRS abnormalities are reported in patients who have potentially reversible causes for bradyarrhythmia. In reversible bradyarrhythmia patients who were on AV-blocking drugs and were treated with a TPI and concomitant cessation of drug therapy, all patients with bifascicular block and BBB and >95% of patients on beta-blocker treatment received permanent pacing (12). Permanent pacing is indicated in patients on beta-blockers and QRS width ≥120 ms who develop high-degree AV block (12, 14). In a recent study, Duarte et al. (3) reported that presentation with advanced AV block had a higher risk of recurrence in patients with iatrogenic or potentially reversible bradyarrhythmia. Similar to our study, univariate analyses showed a significant association between ECG features suggestive of underlying conduction disease (AVND, advanced AV block, QRS duration ≥120 ms, bifascicular block, and BBB) and permanent pacing for a recurrence of bradyarrhythmia (Table 3). However, multivariate regression analysis revealed only advanced AV block at presentation and bifascicular block at discharge as independent predictors of permanent pacing for a recurrence of bradyarrhythmia. Our findings suggest that the recurrence of bradyarrhythmia is dependent on the degree of underlying conduction abnormality. An abnormal, but vulnerable AV conduction system may be prone to manifest with bradycardia, when challenged with potential factors capable of altering conduction characteristics. In a univariate analysis of our study, TPI was more frequently used in patients who had achieved the primary as well as secondary endpoints than in those who did not. Unlike our study, Farre et al. (4) reported no significant association of NSRB with a TPI.

The short follow-up period and selective participant criteria likely contributed to the reduced incidence of cardiovascular events observed. By excluding patients with conditions such as heart failure, coronary artery disease, inflammatory or infectious diseases, as well as those requiring pacing during their initial hospital stay, the study population omitted many high-risk patients. This selective approach may have resulted in the lower rate of cardiovascular events reported.

### Limitations

The present study was a single-center study with inherent limitations and included only symptomatic patients who were hospitalized. Follow-up in our study was symptom-guided, and clinically insignificant bradycardia episodes were not included in the analysis. Hence, these observations may have underestimated the recurrence rate and cannot be generalized to ambulatory or asymptomatic patients. Ambulatory ECG monitoring and longer follow-up studies are needed to identify the recurrence of NSRB. Due to the absence of an electrophysiology study, the exact location of the block could not be determined, potentially resulting in the misclassification of some patients, particularly those with Mobitz type I as advanced AV block. Also, in our study, we relied on serum creatinine alone to assess renal dysfunction rather than the estimated glomerular filtration rate (eGFR). Another limitation of our study is that the cause–effect relationship of the reversible factors and bradyarrhythmia was not established. Moreover, differentiation between spontaneous intermittent bradyarrhythmia, reflex bradyarrhythmia, and bradyarrhythmia due to reversible etiology could not be done. It is difficult to infer from our study whether primary hyperkalemia led to AV block or whether hemodynamic instability led to secondary hyperkalemia.

## Conclusions

The present study shows that patients with NSRB are at a high risk of recurrence within a short follow-up duration. The presence of advanced AV block at presentation and persistent bifascicular block after reversal of bradyarrhythmia was significantly associated with recurrence of NSRB. Our results suggest permanent pacing during index hospitalization in these patients.

## Data Availability

The original contributions presented in the study are included in the article/Supplementary Material, further inquiries can be directed to the corresponding author.
