# Supplementary material for: Recurrence rate and predictors in non-ischemic reversible bradyarrhythmias
Source: Front Cardiovasc Med. 2024 Oct 9;11:1455018. doi: 10.3389/fcvm.2024.1455018 (PMC11496075; doi:10.3389/fcvm.2024.1455018)
Supplement: Supplementary file 1 [file Datasheet1.docx]

Table S1**:** The echocardiographic details observed in the study

| **Echo variables** | **N= 124 patients** |
| --- | --- |
| LVEF, % | 59.75 ± 7.17 |
| RWMA | 16 (12.9%) |
| Valvular disorder | |
| Moderate TR | 3 (2.4%) |
| Moderate As | 1 (0.8%) |
| Rheumatic severe MS | 2 (1.6%) |

*Values are presented as mean ± standard deviation for continuous variables.*

*AS: Aortic stenosis; MS: Mitral stenosis; TR: Tricuspid regurgitation*

## Tables S2: Combined effects of different reversible factors (​Online supplement)

| **Treatments** | **Primary Endpoint** | | **p-value​** |
| --- | --- | --- | --- |
|  | **Reached**  **(n=27)** | **Not reached (n=97)** |  |
| Drugs + Hyperkalemia | 3 (11.1%) | 15 (15.5%) | 0.761 |
| Drugs + Hypothyroidism | 0 (0.0%) | 10 (10.3%) | 0.116 |
| Hyperkalemia + Hypothyroidism | 0 (0.0%) | 3 (3.1%) | 1 |
| Drugs + Hyperkalemia + Hypothyroidism | 1 (3.7%) | 0 (0.0%) | 0.218 |
| >1 Drug | 1 (3.7%) | 10 (10.3%) | 0.453 |
| Renal dysfunction + Hyperkalemia | 12 (44.4%) | 49 (50.5%) | 0.577 |
| >1 Reversible factors | 4 (14.8%) | 28 (28.9%) | 0.481 |

| **Treatments** | **Secondary Endpoint** | | **p-value**​ |
| --- | --- | --- | --- |
|  | **Reached (n=33)** | **Not reached (n=91)** |  |
| Drugs + Hyperkalemia | 5 (15.2%) | 13 (14.3%) | 0.904 |
| Drugs + Hypothyroidism | 0 (0.0%) | 10 (11.0%) | 0.061 |
| Hyperkalemia + Hypothyroidism | 1 (3.0%) | 2 (2.2%) | 1 |
| Drugs + Hyperkalemia + Hypothyroidism | 1 (3.0%) | 0 (0.0%) | 0.266 |
| >1 Drug | 1 (3.0%) | 10 (11.0%) | 0.285 |
| Renal dysfunction + Hyperkalemia | 17 (51.5%) | 44 (48.4%) | 0.755 |
| >1 Reversible factors | 7 (21.2%) | 25 (27.5%) | 0.481 |

## Tables S3: Association of reversible etiological factors with advanced atrioventricular block at presentation and bifascicular block at the time of discharge (Online supplement)

| **Patient characteristics** | **Total (n=38)** | **Advanced AV Block** | | | **p-value** | **Advanced AV Block** | | | **p-value** |
| --- | --- | --- | --- | --- | --- | --- | --- | --- | --- |
|  |  | **PE Reached** | **PE Not Reached** | |  | **SE Reached** | **SE Not Reached** | |  |
|  |  | **(n=20)** | **(n=18)** | |  | **(n=20)** | **(n=18)** | |  |
| **Reversible etiological factors, n (%)** | | | | | | | | | |
| **Rate limiting drugs** | | | | | | | | | |
| Beta-blockers | 21(55.3%) | 13(65.0%) | | 8(44.4%) | 0.203 | 13(65.0%) | | 8(44.4%) | 0.203 |
| CCBs | 1(2.6%) | 1(5.0%) | | 0(0.0%) | 1 | 1(5.0%) | | 0(0.0%) | 1 |
| Antiarrhythmic drugs | 0(0.0%) | 0(0.0%) | | 0(0.0%) | - | 0(0.0%) | | 0(0.0%) | - |
| Combination of drugs | 2(5.3%) | 1(5.0%) | | 1(5.6%) | 1 | 1(5.0%) | | 1(5.6%) | 1 |
| Hyperkalaemia | 21(55.3%) | 10(50.0%) | | 11(61.1%) | 0.492 | 10(50.0%) | | 11(61.1%) | 0.492 |
| Hypothyroidism | 1(2.6%) | 1(5.0%) | | 0(0.0%) | 1 | 1(5.0%) | | 0(0.0%) | 1 |
| Combined reversible factors​ | 4(10.5%) | 3(15.0%) | | 1(5.6%) | 0.606 | 3(15.0%) | | 1(5.6%) | 0.606 |

*AV: Atrioventricular; CCB: calcium-channel blocker; PE: Primary endpoint; SE: Secondary endpoint*

| **Patient characteristics** | **Total (n=13)** | Bifascicular block | | | **p-value** | Bifascicular block | | | **p-value** |
| --- | --- | --- | --- | --- | --- | --- | --- | --- | --- |
|  |  | **PE Reached** | **PE Not Reached** | |  | **SE Reached** | **SE Not Reached** | |  |
|  |  | **(n=10)** | **(n=3)** | |  | **(n=11)** | **(n=2)** | |  |
| **Reversible etiological factors, n (%)** | | | | | | | | | |
| **Rate limiting drugs** | | | | | | | | | |
| Beta-blockers | 11(84.6%) | 9(90.0%) | | 2(66.7%) | 0.423 | 10(90.9%) | | 1(50.0%) | 0.295 |
| CCBs | 0(0.0%) | 0(0.0%) | | 0(0.0%) | - | 0(0.0%) | | 0(0.0%) | - |
| Antiarrhythmic drugs | 1(7.7%) | 0(0.0%) | | 1(33.3%) | 0.231 | 0(0.0%) | | 1(50.0%) | 0.154 |
| Combination of drugs | 1(7.7%) | 0(0.0%) | | 1(33.3%) | 0.231 | 0(0.0%) | | 1(50.0%) | 0.154 |
| Hyperkalaemia | 5(38.5%) | 2(20.0%) | | 3(100%) | 0.035 | 3(27.3%) | | 2(100%) | 0.128 |
| Hypothyroidism | 0(0.0%) | 0(0.0%) | | 0(0.0%) | - | 0(0.0%) | | 0(0.0%) | - |
| Combined reversible factors​ | 3(23.1%) | 1(10.0%) | | 2(66.7%) | 0.108 | 2(18.2%) | | 1(50.0%) | 0.423 |

*CCB: calcium-channel blocker; PE: Primary endpoint; SE: Secondary endpoint*
